# Direct Matrix-Assisted Laser Desorption-Ionisation (MALDI) Mass-Spectrometry Bacteria Profiling for Identifying and Characterizing Pathogens

**Published:** 2009-04

**Authors:** E. N. Ilina

**Affiliations:** 1 Scientific Research Institute of Physical-Chemical Medicine

## Abstract

This study examines the features and limitations of direct Matrix-Assisted Laser Desorption-Ionisation (MALDI) mass-spectrometry profiling of bacterial cells for investigating a microbial population. The optimal laboratory protocol, including crude bacteria lyses by a solution of 50% acetonitrile, 2.5% trifluoroacetic acid, and using α-cyano-4-hydroxy cinnamic acid as the MALDI matrix, has been developed. Two different bacteria species were under investigation, and representative mass spectra from 278 strains of Neisseria gonorrhoeae and 22 strains of Helicobacter pylori have been analyzed. It's known that both bacteria demonstrate a variable degree of polymorphism. For N. gonorrhoeae, the MALDI mass spectra that was collected possessed about 70 peaks, 20 of which were good reproducible ones. In spite of the fact that three peaks were found with differing spectra in some strains, little diversity in the N. gonorrhoeae population was revealed. This fact indicates the prospects in using direct MALDI mass-spectrometry profiling for gonococcus identification. In the case of H. pylori strains, the variety in the collected mass-spectra was shown to be essential. Only five peaks were present in more than 70% of strains, and a single mass value was common for all spectra. While these data call into question the possibility of the reliable species identification of H. pylori using this approach, the intraspecies differentiation of strains was offered. Good association between MALDI profile distributions and the region of strain isolation have been found. Thus, the suggested direct MALDI mass-spectrometry profiling strategy, coupled with special analysis software, seems promising for the species identification of N. gonorrhoeae but is assumed insufficient for H. pylori species determination. At the same time, this would create a very good chance for an epidemiological study of such variable bacteria as H. pylori.

## INTRODUCTION

Modern microbiology and its applied branches require the development of new rapid and precise methods for identifying clinically significant pathogens and for describing their characteristic features such as virulence, antibiotic sensitivity, and strain group. The relative tolerance of Matrix-Assisted Laser Desorption-Ionisation (MALDI) to contamination with salt and other impurities allows one to conduct a direct mass-spectrometry analysis of the microbial cell content (direct profiling) without the fractionation and purification of some components. 

Generally, the considered method involves analyzing the complex mixtures of cell components such as proteins, peptides, lipids, and nucleic acids. However, the matrix formulation applied and parameters of mass-spectra establishment make it possible to register protein molecules that are of great worth, because a cell contains a significant quantity of variable proteins. 

The registrability of mass-spectra, unique and reproducible for families, genera, species, and subspecies of microorganisms, makes it possible to use mass-spectrometry for bacteria identification and typing, which was demonstrated for the first time in 1975 [1]. Despite the fact that a "whole cell" spectrum (without any separation of the cell components) reflects only an insignificant part of the cell proteome, it is demonstrative enough to characterize the cell taxonomic features, which was established for a number of bacteria [2, 3, 4, 5, 6]. It should be noted that this method does not involve the identification of separate microbial proteins and allows the application of a unique mass-profile for characterizing any microorganism on the "fingerprint pattern" principle [7]. Specific features and, at the same time, disadvantages of the considered approach when compared to the traditional methods are as follows: (1) a quite high sensitivity (105-106 of cells or 0.5 μg of cell culture), (2) simple sample preparation, (3) high measurement rate, and (4) the possibility of automating and robotizing all investigation stages. 

This study examines the advantages and disadvantages of using MALDI mass spectrometry to profile bacterial cells using such microbial populations as Helicobacter pylori and Neisseria gonorrhoeae, which are commonly referred to as microorganisms with a high genetic flexibility of genomes, as examples. Moreover, we set the task of estimating the variability of MALDI mass-profiles which were photographed according to the developed protocol within each bacterial population. 

## METHODS

Bacterial strains The following laboratory strains were used in the investigation: Escherichia coli DH5α, Neisseria gonorrhoeae ATCC 49226, Helicobacter pylori J99, and Helicobacter pylori 26695; 278 N. Gonorrhoeae clinical strains from different regions of Russia (Moscow, St. Petersburg, Samara, Ekaterinburg, Murmansk, and Irkutsk); and 22 H. pylori clinical strains from Mongolia, Tuva, Yakutia, and the Moscow Region.

Fresh cultures of the stationary growth phase produced by the standard microorganism cultivation methods were used in the mass-spectrometry analysis. 

Preparing samples for mass-spectrometry analysis The lysis of bacterial cells involved the trituration of the bacteria culture (single colony) in 50 mcl of 50% acetonitrile (ACN, Sigma-Aldrich, Germany) and 2.5% trifluoroacetic acid (TFA, Sigma-Aldrich, Germany). Supernatant produced in the course of the following centrifugation (1 min at 14,000 r/min) was used for MALDI mass-spectrometry analysis. The saturated solution of α-cyano-4-hydroxycinnamic acid mixed with 50% CAN and 2.5 TFA acted as a matrix (α-CHCA, Bruker Daltoniks, Germany). All the chemical agents that were applied were absolutely pure or meant exclusively for mass-spectrometry analysis. 

Mass-spectrometry analysis The mass-spectrometry analysis was carried out on a time-of-flight MicroflexTM MALDI mass spectrometer (Bruker Daltonics, Germany) equipped with a 337-nm nitrogen laser. All measurements were performed in linear conditions with the detection of positive ions. To accumulate the mass spectra, the laser radiation power was set to the level of the minimum threshold limit value, which was sufficient for the desorption and ionization of the sample. The optimal mass-spectrometer parameters were set in the m/z 2,000-20,000 range. External calibration was based on the precise mass values of well-known E. coli proteins. The sample was applied to three cells in the plate. The spectrum obtained after summing up 10 spectral series per 50 laser bursts was recorded for each cell. The software of the Bruker Daltonics Company (Germany)—flexControl 2.4 (Build 38) and flexAnalysis 2.4 (Build 11)—were used for recording, processing, and analyzing the mass spectra. The accuracy of the mass measurements attained was ±2 Da. 

Interpretation of mass-spectra The interpretation process assumed that most registered peaks corresponded to the protein molecules and the masses determined were the masses of non-fragmented proteins. Proteins were identified by comparing the experimental protein masses with the protein mass values annotated in the corresponding databases (SwissProt/TrEMBL) using the ExPASy-server resources (http://ca.expasy.org/srs5/). The experimental mass value, measured with an accuracy of up to ±2, was used for setting the "Molecular weight" parameter. If the attempt was unsuccessful, we carried out a second search with a mass value corresponding to the loss of N-tail methionine, taking into consideration the posttranslation protein modifications. 

Statistical data processing The program resources of Microsoft Office Excel 2003 were used for creating intermediate tables, performing elementary calculations and descriptive statistics, and constructing diagrams. The cluster analysis was carried out with Statistica 6.0. 

## RESULTS AND DISCUSSION

The protocol of direct MALDI mass-spectrometry bacteria profiling was optimized using the laboratory E. coli DH5α strain, whose protein and nucleotide sequences were studied thoroughly. The method was based on the acid lysis of bacterial cells, which causes the extraction of the major ribosomal proteins that make up 20% of the total pool of E. coli proteins [8].

Solvent systems with different ACN and TFA concentrations and ratios, as well as the three most ubiquitous matrices used for sample ionization in MALDI mass-spectrometry analysis, were tested in the course of experiments to obtain the cell lysate. Estimation criteria were as follows: the reproducibility of MALDI mass-profiles within one strain, the resolution of mass-spectra peaks, the signal/noise ratio, the number of peaks (the representativeness of mass-spectrum), their intensity, and the range of m/z values registered in the course of analysis. The optimal solution (50% ACN, 2.5% TFA) for the lysis of bacterial cells was chosen on the basis of experimental data for further stages of investigation. This solution was suggested to help in creating the most qualitative mass spectra using any matrix substance involved in the investigation.

The chosen conditions of direct MALDI mass-spectrometry profiling allowed us to obtain an E. Coli spectrum analogous in qualitative composition to the spectrum obtained earlier for the E. coli K-12 strain [9] (except for some peaks). This inconsistency is quite logical, because we analyzed another E. Coli strain: namely, DH5α. 

**Fig. 1.**
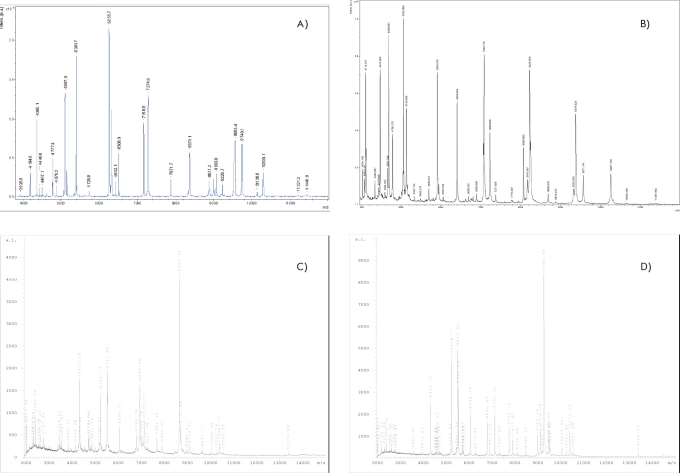
MALDI mass spectra of E. coli DH-5α (A), N. gonorrhoeae ATCC 49226 (B), H. pylori J99 (C), and H. pylori 26695 strains registered using the α-CHCA matrix.

Typical MALDI mass spectra of the laboratory E. coli DH5α, N. gonorrhoeae ATCC 49226, H. pylori J99, and H. pylori 26695 strains that were obtained according to the developed protocol are presented in Fig. 1. Though the mass spectra were established in the m/z 2000-20 000 range, the m/z 2000-12 000 area was the most informative from a visual standpoint. Moreover, a detailed analysis of the mass spectra showed that peaks on the m/z 2000-4000 area corresponded to the divalent ions of molecules whose monovalent ions were registered in the same spectra. Therefore, special attention was paid to the MALDI mass-spectrum area in the m/z 4000-12 000 range. As a whole, the peak list obtained for the laboratory E. coli DH5α strain corresponds to that obtained previously for the coli K-12 strain [9]. 

During each measurement, about 70 peaks were registered for the laboratory N. gonorrhoeae ATCC 49226 strain, 20 of which were easily reproducible. The identification procedure established that 14 of those peaks corresponded to gonococcus ribosomal proteins (Table 1). Analogous data were obtained for the laboratory H. pylori J99 and H. pylori 26695 strains: 14 out of 20 reproducible proteins were ribosomal. 

**Table 1:** The most reproducible peaks of laboratory N. gonorrhoeae ATCC 49226 strain mass-spectrum. Peaks, which are suggested to correspond to ribosomal proteins, are displayed in bold type

№	M (m/z)	M^1^ (Dа)	Ion type^2^	Description
1	4474	4473	M+H^+^	RL36
2	4511			
3	4689	9377^3^	M+2H^+^	RS20
4	4784	9570	M+2H^+^	RL27
5	5010			
6	5052	5051	M+H^+^	RL34
7	5130	10259^3^	M+2H^+^	RS19
8	5484			
9	5908	5907	M+H^+^	RL33
10	5946			
11	6053			
12	6404	6402^3^	M+H^+^	RL32
13	7080	7078	M+H^+^	RL29
14	7227	7226^3^	M+H^+^	RL35
15	8068			
16	8167	8165	M+H^+^	RL31
17	8225	8224^3^	M+H^+^	RS21
18	9379	9377^3^	M+H^+^	RS20
19	9570	9568^3^	M+H^+^	RL27
20	10260	10259^3^	M+H^+^	RS19

^1^ Protein mass annotated in the SwissProt/TrEMBL databases ^2^ Ion types are indicated only for peaks that correspond to certain proteins ^3^ Protein mass with loss of N-tail methionine

Comparing the H. pylori J99 and 26695 strains spectra revealed some differences (displacement and presence or absence of peaks) (Fig. 1, Table 2). Differences in peaks regarding ribosomal proteins may be explained on the basis of annotation to the genomes of these two strains, in particular, taking into account the data on amino-acid sequences of the corresponding proteins (RL32, RL29, RL24, RS16). Other differences of the spectrum peaks defy explanations at this level of investigation. 

The developed methods for obtaining the reproducible MALDI mass-profiles of bacterial strains allowed us to analyze geographically heterogeneous groups of such clinical strains as gonococcus and H. pylori. 

**Table 2:** The most reproducible peaks of the mass spectra of laboratory H. pylori J99 and H. pylori ATCC 26695 strains. Peaks, which are suggested to correspond to ribosomal proteins, are displayed in bold type

H. pylori J99		H. pylori ATCC 26695		M1 (Dа)	Ion types2	Description
№	M (m/z)	№	M (m/z)			
1	4322	1	4322	4320	M+H^+^	RL36
2	5247	2	5247	5246	M+H^+^	RL34
3	5517			5515	M+H^+^	RL32
		3	5530	5529	M+H^+^	RL32
4	5541	4	5541			
5	6068	5	6068	6066	M+H^+^	RL33
6	6799	6	6799	6798^3^	M+H^+^	RL28
7	6912	7	6912			
8	6947	8	6947	6946	M+H^+^	Hpn^4^
9	7130	9	7130	7129^3^	M+H^+^	RL35
10	7654	10	7654	7652	M+H^+^	RL31
		11	7684	7683	M+H^+^	RL29
11	7753			7752	M+H^+^	RL29
		12	7906	7905	M+H^+^	RL24
12	7917			7915	M+H^+^	RL24
13	8484	13	8484	8482	M+H^+^	RS21
		14	8657			
14	8972			8971	M+H^+^	RS16
		15	8986	8985	M+H^+^	RS16
		16	9114			
15	9129					
16	10067	17	10067	10065	M+H^+^	RS20
17	10260	18	10260			
		19	10384			
18	10414					
19	10450	20	10450	10448	M+H^+^	RS18
		21	10544	10543	M+H^+^	RS19
20	10557					

^1^ Protein mass annotated in the SwissProt/TrEMBL databases ^2^ Ion types are indicated only for peaks that correspond to certain proteins ^3^ Protein mass with loss of N-tail methionine ^4^ Histidine-rich metal-binding polypeptide (Hpn), which is known to bind Ni^2+^ and Zn^2+^, but its function in the bacterial cell's vital activity is still unclear [14].

We carried out the MALDI mass-spectrometry profiling of 278 N. gonorrhoeae clinical strains taken from different regions of Russia. According to a comparative analysis of the mass spectra (peak lists) obtained, three peaks have the following m/z values: 4473, 5051, and 8165. These values correspond to such ribosomal proteins as RL36, RL34, and RL31, according to the N. gonorrhoeae ATCC 49226 mass-spectrum, and can occasionally vary to 4487, 5081, and 8146, respectively. Four combinations of variable m/z values of those proteins were revealed among the group of strains investigated. Taking into account the invariability of other m/z values in the spectrum, we distinguished four types of MALDI mass profiles (prototypes) of gonococcus. The investigated gonococcus strains (n = 278) were distributed as follows: 236 strains (84.9%) corresponded to type 1 (m/z 4473 / 5051 / 8165), i.e., control strain of N. gonorrhoeae ATCC 49226; 26 strains (9.4%) were referred to type 2 (m/z 4487 / 5051 / 8165); 15 strains (5.4%), to type 3 (m/z 4487 / 5051 / 8147); and 1 strain from Irkutsk (0.4%), to type 4. Simpson's diversity index [10] for typing with the direct mass-spectrometry profiling of the gonococcus strain collection was 0.27. 

**Fig. 2.**
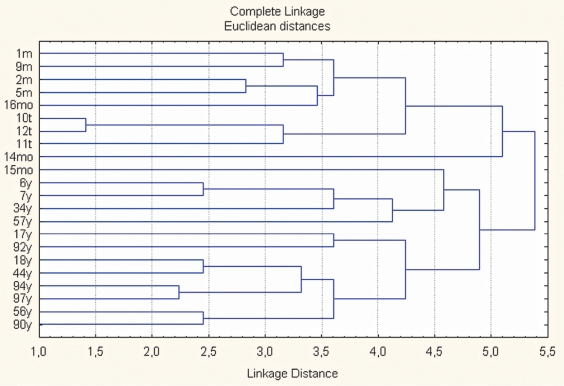
Distribution of H. Pylori strains from Mordovia (m), Tuva (t), Yakutia (y), and Moscow Region (mo) according to the results of a cluster analysis of MALDI mass-profiles.

Twenty-two H. pylori clinical strains recovered in different regions of Russia (Yakutia, Tuva, Mordovia, and Moscow oblast) were subject to typing. A comparative analysis revealed numerous variations and differences in those strains. If the registered peak displacement is suggested to be a variation in the m/z values, which group around a certain average value, and to reflect the mass and structure change of the same protein, it may be concluded that only 2 out of 20 spectrum proteins retain a constant mass in the group investigated. Nevertheless, the cluster analysis of the peak lists that were obtained allowed us to group the strains studied into three big groups correlating to the geographical specificity of the strains (Fig. 2). Taking into consideration the small volume of the sample collection (less than 30 samples), the Simpson's diversity index was not estimated. 

In spite of the rich mass spectra with up to 70 peaks, direct MALDI mass-spectrometry profiling of laboratory (n = 1) and clinical (n = 278) N. gonorrhoeae strains revealed the homogeneity of profiles in the group investigated. It should be noted that the MALDI mass spectra photographed for gonococcus according to the protocol developed are much richer than those annotated previously [11]. Insignificant differences characteristic for the mass-spectra qualitative composition of clinical gonococcal strains are consistent with our ideas about the high conservatism of ribosomal proteins. 

The low heterogeneity of the mass-spectra qualitative composition makes this approach inappropriate for gonococcus typing. However, these results have a reverse side and they establish the stability of the mass-spectra qualitative composition, which offers great opportunities for the species identification of a causative agent using this method. The marker mass profile composed of 20 stable displayable peaks may be used for identifying an individual species by comparing it with the experimental mass-spectrum of an unknown microorganism. 

On the contrary, the direct MALDI mass-spectrometry profiling of laboratory (n = 2) and clinical (n = 22) H. pylori strains demonstrated a well-pronounced heterogeneity of the mass profiles obtained. Both qualitative and quantitative compositions varied. The mass spectra of laboratory strains were quite rich (up to 30 peaks), whereas the mass spectra of the clinical strains were rather poor (7-13 peaks). Similar results were presented in previous investigations [12, 13], which testifies to the regularity of this phenomenon. Only five peaks remained unchanged in the mass profiles of the strains investigated. Four of them are supposed to correspond to ribosomal proteins, and the fifth is referred to as a histidine-rich metal-binding polypeptide (Hpn). Hpn is known to bind Ni^2+^ and Zn^2+^, but its function in the bacterial cell's vital activity is still unclear [14]. 

It should be noted that significant variability within one species is consistent with such well-known characteristic features of Helicobacter as the expressed macro- and microheterogeneity of the genome [15, 16, 17]. Establishing intraspecies heterogeneity by this method gives grounds to assume that this approach may be used for the same intraspecies classification and typing of microorganisms that was demonstrated with the help of cluster-analysis means. 

## CONCLUSIONS

The consolidated experience of using direct MALDI mass-spectrometry bacteria profiling gives grounds to assume that this approach may be used for the specific identification of the causative agent that was demonstrated when analyzing the voluminous group of clinical N. gonorrhoeae strains. On the contrary, intraspecies classification and typing of bacteria with this method is likely to be uninformative for species with a low intraspecies variability. 

On the other hand, identifying bacteria characterized by a high intraspecies variability, for instance, H. pylori, is still a matter of debate and requires additional study. The data obtained demonstrate prospects for using direct MALDI mass-spectrometry profiling for strain differentiation and bacteria typing. 

## Acknowledgements

The work was supported by the Bruker Daltonics Company, Germany (grant no. 270505(#30686).
